# Semantic units: organizing knowledge graphs into semantically meaningful units of representation

**DOI:** 10.1186/s13326-024-00310-5

**Published:** 2024-05-27

**Authors:** Lars Vogt, Tobias Kuhn, Robert Hoehndorf

**Affiliations:** 1 TIB Leibniz Information Centre for Science and Technology, Welfengarten 1B, 30167 Hanover, Germany; 2 Department of Computer Science, Vrije Universiteit, Amsterdam, Netherlands; 3 Computational Bioscience Research Center, Computer, Electrical and Mathematical Sciences & Engineering Division, King Abdullah University of Science and Technology, 4700 KAUST, 23955 Thuwal, Saudi Arabia

**Keywords:** FAIR data and metadata, Knowledge graph, OWL, RDF, Semantic unit, Graph organization, Granularity tree, Representational granularity

## Abstract

**Background:**

In today’s landscape of data management, the importance of knowledge graphs and ontologies is escalating as critical mechanisms aligned with the FAIR Guiding Principles—ensuring data and metadata are Findable, Accessible, Interoperable, and Reusable. We discuss three challenges that may hinder the effective exploitation of the full potential of FAIR knowledge graphs.

**Results:**

We introduce “semantic units” as a conceptual solution, although currently exemplified only in a limited prototype. Semantic units structure a knowledge graph into identifiable and semantically meaningful subgraphs by adding another layer of triples on top of the conventional data layer. Semantic units and their subgraphs are represented by their own resource that instantiates a corresponding semantic unit class. We distinguish statement and compound units as basic categories of semantic units. A statement unit is the smallest, independent proposition that is semantically meaningful for a human reader. Depending on the relation of its underlying proposition, it consists of one or more triples. Organizing a knowledge graph into statement units results in a partition of the graph, with each triple belonging to exactly one statement unit. A compound unit, on the other hand, is a semantically meaningful collection of statement and compound units that form larger subgraphs. Some semantic units organize the graph into different levels of representational granularity, others orthogonally into different types of granularity trees or different frames of reference, structuring and organizing the knowledge graph into partially overlapping, partially enclosed subgraphs, each of which can be referenced by its own resource.

**Conclusions:**

Semantic units, applicable in RDF/OWL and labeled property graphs, offer support for making statements about statements and facilitate graph-alignment, subgraph-matching, knowledge graph profiling, and for management of access restrictions to sensitive data. Additionally, we argue that organizing the graph into semantic units promotes the differentiation of ontological and discursive information, and that it also supports the differentiation of multiple frames of reference within the graph.

## Background

In an era marked by the exponential generation of data [1–3], both technically and socially intricate challenges have emerged [4], necessitating innovative approaches to data representation and management in science and industry. The growing volume of data production calls for systems capable of collecting, integrating, and analyzing extensive datasets from diverse sources, a critical requirement in addressing contemporary global challenges [5]. Notably, data stewardship should rest within the hands of the domain experts or institutions to ensure technical autonomy, aligning with the concept of *data visiting* rather than conventional *data sharing* [6].

From the standpoint of data management and representation, meeting these demands relies on adherence to the **FAIR Guiding Principles**—enabling data and metadata to be readily **F**indable, **A**ccessible, **I**nteroperable, and **R**eusable for machines and humans alike [7]. Failure to achieve FAIRness risks transforming Big Data into opaque Dark Data [8]. Establishing the FAIRness of data and metadata not only contributes to a solution for the reproducibility crisis in science [9] but also addresses broader concerns regarding the trustworthiness of information (see also the **TRUST** Principles of **T**ransparency, **R**esponsibility, **U**ser Focus, **S**ustainability, and **T**echnology [10]).

To capitalize on the transformative potential of the FAIR Principles, the idea of an Internet of FAIR Data and Services was suggested [11]. It should seamlessly scale with the demands of Big Data, enabling relevant data-rich institutions, research projects, and citizen-science initiatives to make their data and metadata universally accessible in adherence to the FAIR Guiding Principles [12, 13]. The key lies in furnishing comprehensive, machine-actionable^1^ data and metadata, complemented by human-readable interfaces and search capabilities.

**Knowledge graphs** can contribute to the needed technical frameworks, offering a structure for managing and representing FAIR data and metadata [14]. Knowledge graphs are particularly applied in the context of semantic search based on entities and relations, deep reasoning, disambiguation of natural language, machine reading, and entity consolidation for Big Data and text analytics [15].

The distinctive graph-based abstractions inherent in knowledge graphs yield advantages over traditional relational or other NoSQL models. These include (i) an intuitive way for modelling relations, (ii) the flexibility to defer data schema definitions to accommodate evolving knowledge, which is especially important when dealing with incomplete knowledge, (iii) incorporation of machine-actionable knowledge representation formalisms like ontologies and rules, (iv) deployment of graph analytics and machine learning, and (v) utilization of specialized graph query languages that support, in addition to standard relational operators such as joins, unions, and projections, also navigational operators for recursively searching for entities through arbitrary-length paths [16–22]. Moreover, the inherent semantic transparency of knowledge graphs can improve the transparency of data-based decision-making and improve the communication of data and knowledge within research and science in general [23–27].

Despite offering an appropriate technical foundation, the utilization of a knowledge graph for storing data and metadata does not inherently ensure the achievement of the FAIR Guiding Principles. Realizing FAIR data and metadata necessitates adherence to specific guidelines, encompassing the consistent application of adequate semantic data models tailored to distinct types of data and metadata statements. This approach is pivotal for ensuring seamless interoperability across a dataset.

In the *Problem statement* section, we discuss three specific challenges that, from our perspective, can be effectively addressed by systematically organizing a knowledge graph into well-defined subgraphs. Prior attempts at this, such as defining a characteristic set as a subgraph based on triples that share the same resource in the *Subject* position, have demonstrated noteworthy enhancements in space and query performance [28, 29] (see also the related concept of RDF molecules [30, 31]), but they do not fully mitigate the challenges outlined below.

**Fig. 1:**
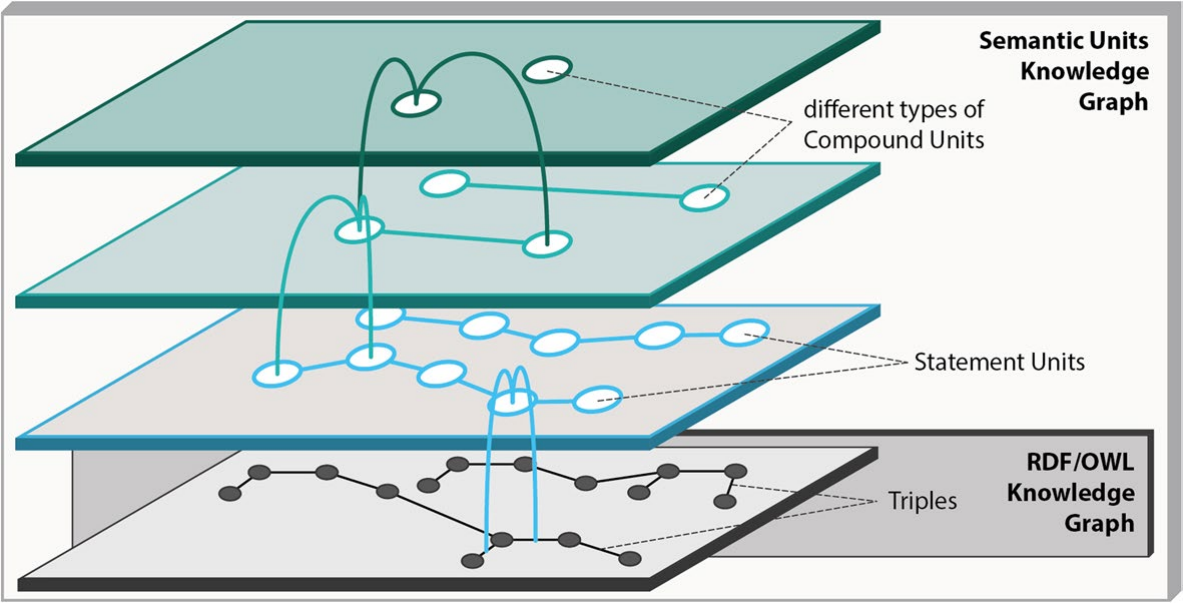
Semantic units introduce additional layers atop the RDF/OWL layer of triples within a knowledge graph. The figure illustrates a partitioning of the triple layer into statement units, wherein each triple aligns with exactly one statement unit, and each statement unit contains one or more triples. Statement units can be organized into diverse types of semantically meaningful collections, denoted as compound units. Compound units serve as the basis for defining several layers that contribute to the enhanced structuring and organization of the knowledge graph in semantically meaningful ways

The *Results* section introduces a novel concept—the partitioning and structuring of a knowledge graph into **semantic units**, identifiable subgraphs represented in the graph with their **own resource**. Semantic units are **semantically meaningful units of representation**, which will contribute to overcoming the challenges at hand. The concept builds upon an idea originally proposed for structuring descriptions of phenotypes into distinct subgraphs, each of which models a descriptive statement like a particular weight measurement or a particular parthood statement for a given anatomical entity [32]. Each such subgraph is organized in its own Named Graph and functions as a smallest semantically meaningful unit in a phenotype description. Generalizing and extending this concept, we present semantic units as accessible, searchable, identifiable, and reusable data items in their own right, forming units of representation implemented through graphs based on the Resource Description Framework (RDF) and the Web Ontology Language (OWL) or labeled property graphs. Two basic categories of semantic units—statement units and compound units—are introduced, supplementing the well-established triples and the overall graph in FAIR knowledge graphs. These units offer a structure that organizes a knowledge graph into five levels of representational granularity, from individual triples to the graph as a whole. In further refinement, additional subcategories of semantic units are proposed for enhanced graph organization. The incorporation of Unique Persistent and Resolvable Identifiers (UPRIs) for each semantic unit enables their efficient referencing within triples, facilitating an efficient way of making statements about statements. The introduction of semantic units adds further layers of triples to the well-established RDF and OWL layer for knowledge graphs (Fig. 1). This augmentation aims to enhance the usability of knowledge graphs for both domain-experts and developers.

In the *Discussion* section, we discuss the benefits we see from organizing knowledge graphs into distinct knowledge graph modules (i.e., semantic units) in terms of increasing data management flexibility and explorability of the graph. We also discuss possible strategies for implementing semantic units for RDF/OWL-based and labeled-property-graph-based knowledge graphs. Table 1.

**Table 1:** Conventions

In this paper, the term *knowledge graph* denotes a machine-actionable semantic graph employed for the documentation, organization, and representation of data and metadata. It is essential to note that our discussion of semantic units is situated within the context of RDF-based triple stores, OWL, and Description Logics serving as a formal framework for inferencing, alongside labeled property graphs as an alternative to triple stores. We deliberately focus on these technologies as they constitute the primary technologies and logical frameworks within the knowledge graph domain, benefiting from widespread community support and established standards. We are aware of the fact that alternative technologies and frameworks exist that support an n-tuples syntax and more advanced logics (e.g., First Order Logic) [33, 34], but supporting tools and applications are missing or are not widely used to turn them into well-supported, scalable, and easily usable knowledge graph applications. Throughout this text, regular underlining is employed for indicating ontology classes, while *italicsUnderlined* text is reserved for referencing properties. Identification (ID) numbers, formed by the ontology prefix followed by a colon and a number, uniquely specify each resource (e.g., *isAbout* (IAO:0000136)). When a term is not yet covered in any ontology, we denote the corresponding class with an asterisk (*). New classes and properties that relate to semantic units will use the ontology prefix SEMUNIT as in the class *SEMUNIT:metric measurement statement unit*. These will be part of a future Semantic Unit ontology. We use ‘regular underlined’ to indicate instances of classes, with the label referring to the class label and the ID to the ID of the class. The term *resource* is employed to signify something uniquely designated, such as a Uniform Resource Identifier (URI), about which informative statements are made. It thus stands for something and represents something you want to talk about. In RDF, the *Subject* and the *Predicate* in a triple are always resources, whereas the *Object* can be either a resource or a literal. Resources encompass properties, instances, and classes, with properties occupying the *Predicate* position in a triple, instances referring to individuals (=particulars), and classes representing universals or kinds. To maintain clarity, resources are represented with human-readable labels in both the text and all figures, opting for the implicit assumption that each property, instance, and class possesses its UPRI. Additionally, the term *triple* refers specifically to a triple statement, while *statement* pertains to a natural language statement, establishing a clear distinction between the two.	

## Methods

### Problem statement

#### Challenge 1: Ensuring schematic interoperability for FAIR empirical data

In the pursuit of FAIRness in empirical data and metadata in a knowledge graph, it is important not only for the terms employed in data and metadata statements to possess identifiers from controlled vocabularies, such as ontologies, ensuring terminological interoperability, but also the **semantic graph patterns** underlying each statement. These patterns specify the relationships among the terms in a statement, facilitating **schematic interoperability**.

**Fig. 2:**
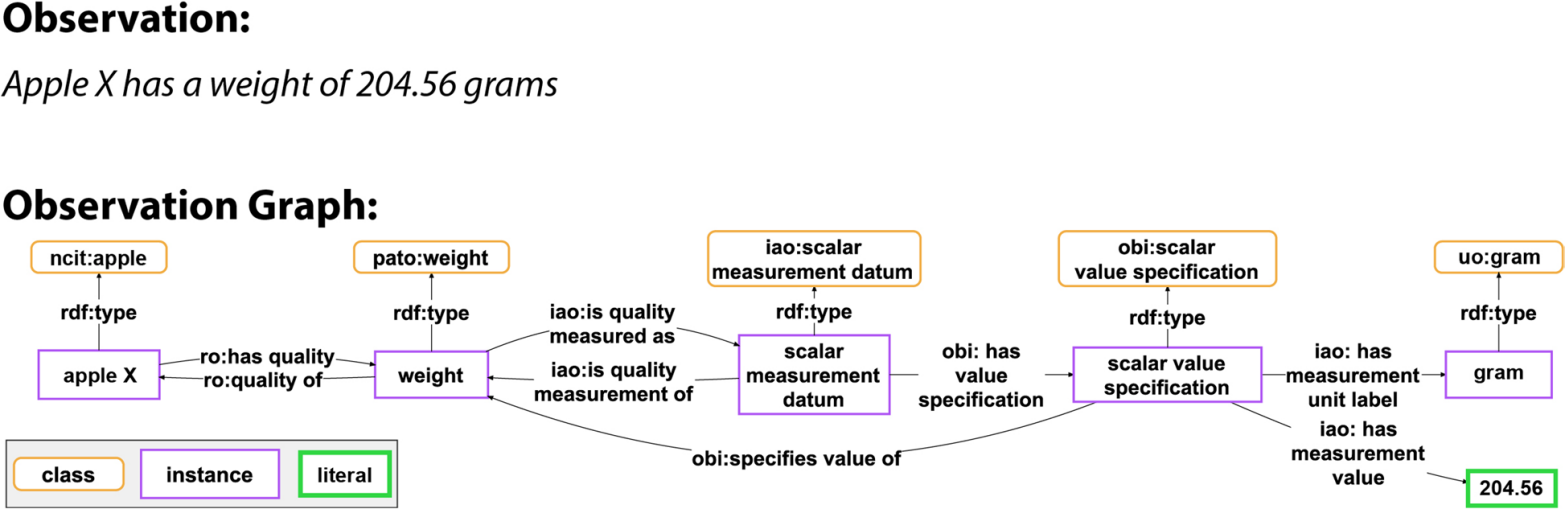
Comparison of a human-readable statement with its machine-actionable representation as a semantic graph following the RDF syntax. Top: A human-readable statement concerning the observation that a specific apple (X) weighs 204.56 grams. Bottom: The corresponding representation of the same statement as a semantic graph, adhering to RDF syntax and following the established pattern for measurement data from the Ontology for Biomedical Investigations (OBI) [35] of the Open Biological and Biomedical Ontology Foundry (OBO)

**Fig. 3:**
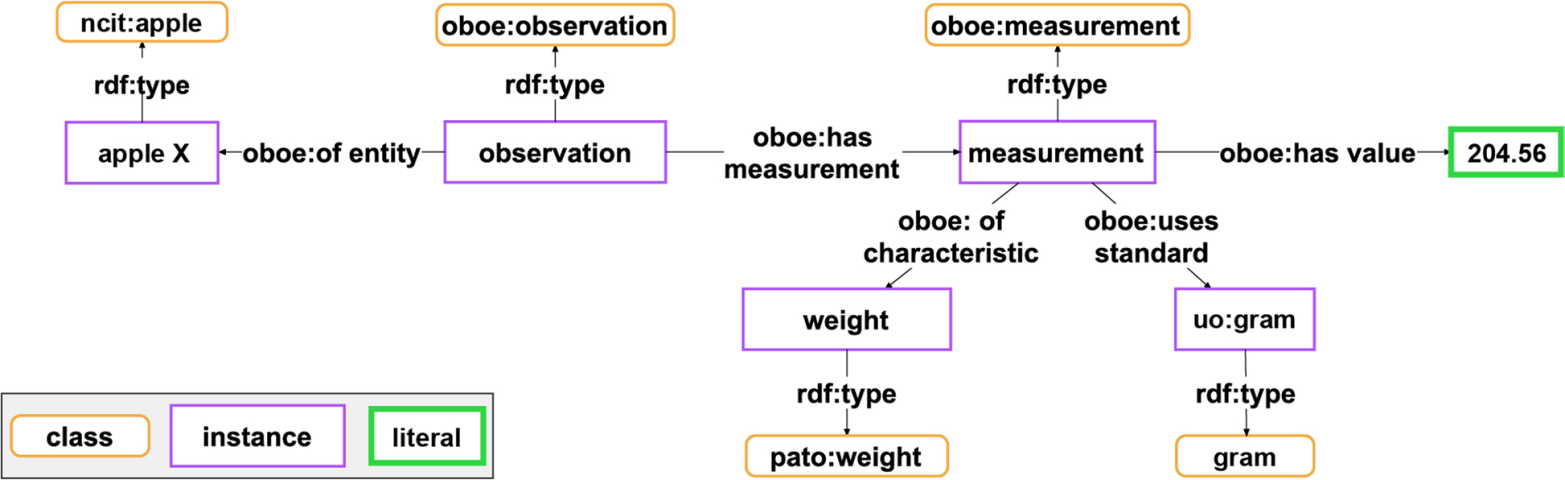
Alternative machine-actionable representation of the data statement from Fig. 2. This graph represents the same data statement as shown in Fig. 2 Top, but applies a semantic graph model that is based on the Extensible Observation Ontology (OBOE) [36], an ontology frequently used in the ecology community

Due to the expressivity of RDF and OWL, statements can be modelled in multiple, often not directly interoperable ways within a knowledge graph. Distinguishing between RDF graphs with different structures that essentially model the same underlying data statement poses a challenge. Consequently, the presence of schematic interoperability conflicts becomes unavoidable, especially when data are represented using diverse graph patterns (cf. Figs. 2 and 3).

Therefore, to maintain interoperability in the representation of empirical data statements within an RDF graph, it can be beneficial to restrict the graph patterns employed for their semantic modelling. Statements of the same type, such as all weight measurements, would employ identical graph patterns to maintain interoperability. Each of these patterns would be assigned an **identifier**. When representing empirical data in the form of an RDF graph, the graph’s metadata should reference that graph-pattern identifier. This approach enables the identification of potentially interoperable RDF graphs sharing common graph-pattern identifiers.

Practically implementing these principles entails two criteria. Firstly, all statements within a knowledge graph must be categorized into statement classes, each associated with a specified graph pattern, typically in the form of a shape specification. Secondly, the subgraph corresponding to a particular statement must be distinctly identifiable.

#### Challenge 2: Overcoming barriers in graph query language adoption

Another significant challenge arises in the context of searching for specific information in a knowledge graph. The prevalent formats for knowledge graphs include RDF/OWL or labeled property graphs like Neo4j. Interacting directly with these graphs, encompassing CRUD operations for creating (= writing), reading (= searching), updating, and deleting statements in the knowledge graph, necessitates the utilization of a query language. SPARQL [37] is an example for RDF/OWL, while Cypher [38] is employed for Neo4j.

Although these query languages empower users to formulate detailed and intricate queries, the challenge lies in their complexity, creating an entry barrier for seamless interactions with knowledge graphs [39]. Furthermore, query languages are not aware of graph patterns.

This challenge may potentially be addressed by providing reusable query patterns that link to specific graph patterns, thereby integrating representation and querying.

#### Challenge 3: Addressing complexities in making statements about statements

**Fig. 4:**
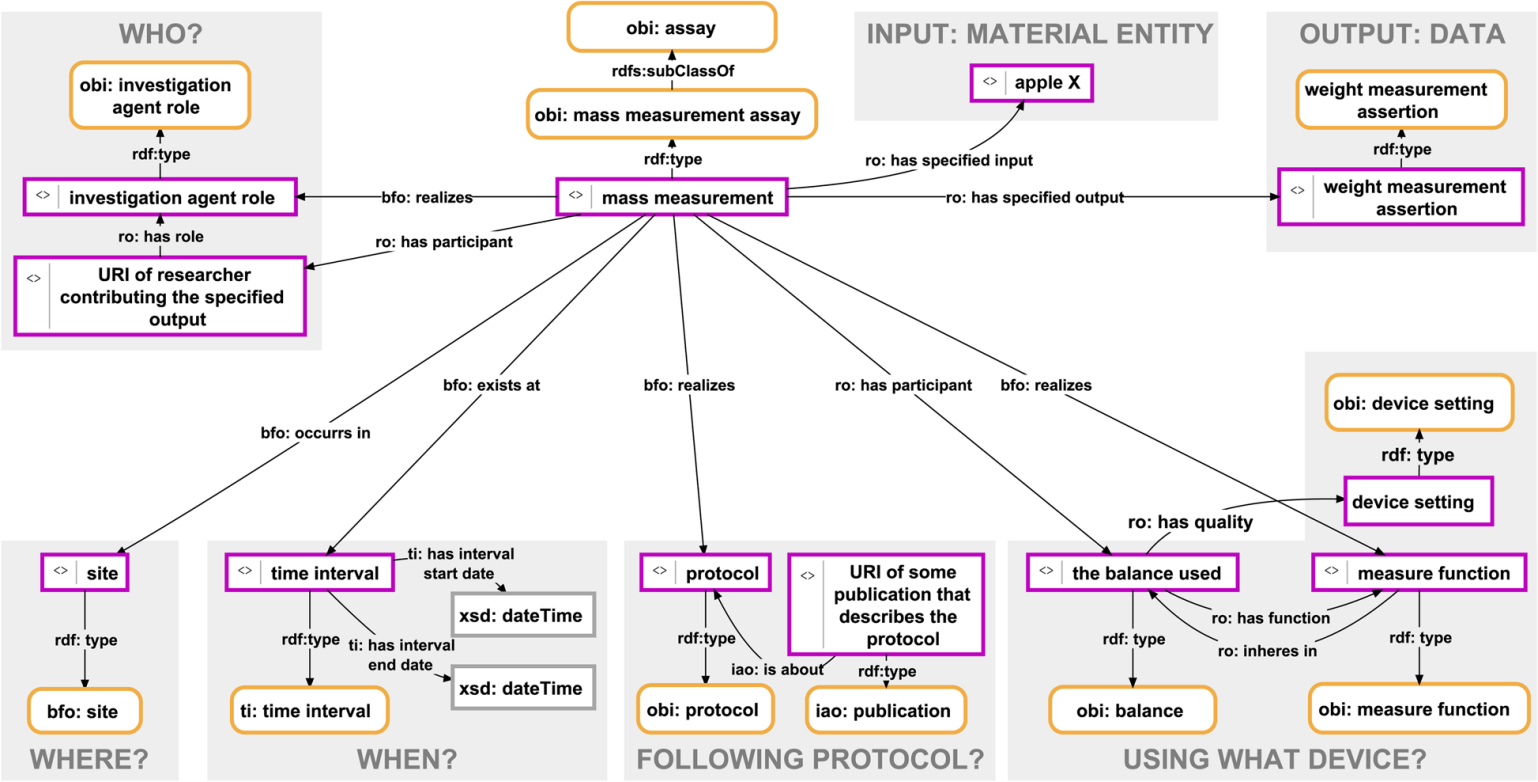
A detailed machine-actionable representation of the metadata relating to a weight measurement datum. This detailed illustration presents a machine-actionable representation of a mass measurement process employing a balance. It documents metadata associated with a weight measurement datum, articulated as an RDF graph. The graph establishes connections between an instance of mass measurement assay (OBI:0000445) and instances of various other classes from diverse ontologies. Noteworthy details include the identification of the measurement conductor, the location and timing of the measurement, the protocol followed, and the specific device utilized (i.e., a balance). Additionally, the graph outlines the material entity serving as the subject and input for the measurement process (i.e., ‘*apple X*’), along with specifying the resultant data encapsulated in a particular weight measurement assertion

The RDF triple syntax of *Subject*, *Predicate*, and *Object* allows expressing a statement about another statement by creating a triple that relates a statement, composed of one or more triples, to a value, resource, or another statement. The scenario may arise where such statements about statements must be modelled. For instance, metadata for a measurement may relate two distinct subgraphs: one representing the measurement itself (as seen in Fig. 2) and another documenting the underlying measuring process (as seen in Fig. 4).

In RDF reification, a statement resource is defined to represent a particular triple by describing it via three additional triples that specify its *Subject*, *Predicate*, and *Object*. Alternatively, the RDF-star approach can be employed [40, 41]. Both methods increase complexity of the represented graph.

In cases like this, the adoption of Named Graphs is an alternative compared to RDF reification or RDF-star approaches. Within RDF-based knowledge graphs, a Named Graph resource identifies a set of triples by incorporating the URI of the Named Graph as a fourth element to each triple, transforming them into quads. In labeled property graphs, on the other hand, assigning a resource for identifying subgraphs within the overall data graph is straightforward and can be achieved by incorporating the resource identifier as the value of a corresponding property-value pair, subsequently adding this pair to all relations and nodes belonging to the same subgraph.

## Results

### Semantic unit

We developed an approach for organizing knowledge graphs into distinct layers of subgraphs using graph patterns. Unlike traditional methods of partitioning a knowledge graph that (i) rely on technical aspects such as shared graph-topological properties of its triples with the goal of (federated) reasoning and query optimization (see *characteristic sets* [29, 30], *RDF molecules* [31, 42], and other approaches [43–45]), that (ii) partition a knowledge graph into small blocks for embedding and entity alignment learning to scale knowledge graph fusion [46], or that (iii) partition knowledge extractions, allowing reasoning over them in parallel to speed up knowledge graph construction [47], our approach introduces semantic units. **Semantic units** prioritize structuring a knowledge graph into **identifiable sets of triples**—subgraphs that represent **units of representation possessing semantic significance for human readers**. Technically, a semantic unit is a **subgraph** within a knowledge graph, represented in the graph by its **own resource**—designated as a UPRI—and embodied in the graph as a node. This resource is classified as an **instance of a specific semantic unit class**.

Semantic units focus on creating units that are semantically meaningful to domain experts. For instance, the graph in Fig. 2 exemplifies a subgraph that can be organized in a semantic unit that instantiates the class *SEMUNIT:weight statement unit* as it is illustrated in Fig. 6. The statement unit models a single, human-readable statement, as opposed to the individual triple ‘weight’ (PATO:0000128) *isQualityMeasuredAs* (IAO:0000417) ‘scalar measurement datum’ (IAO:0000032), which is a single triple from that subgraph. That triple, without the context of the other triples in the subgraph, lacks semantic meaningfulness for a domain expert who has no background in semantics.

**Fig. 5:**
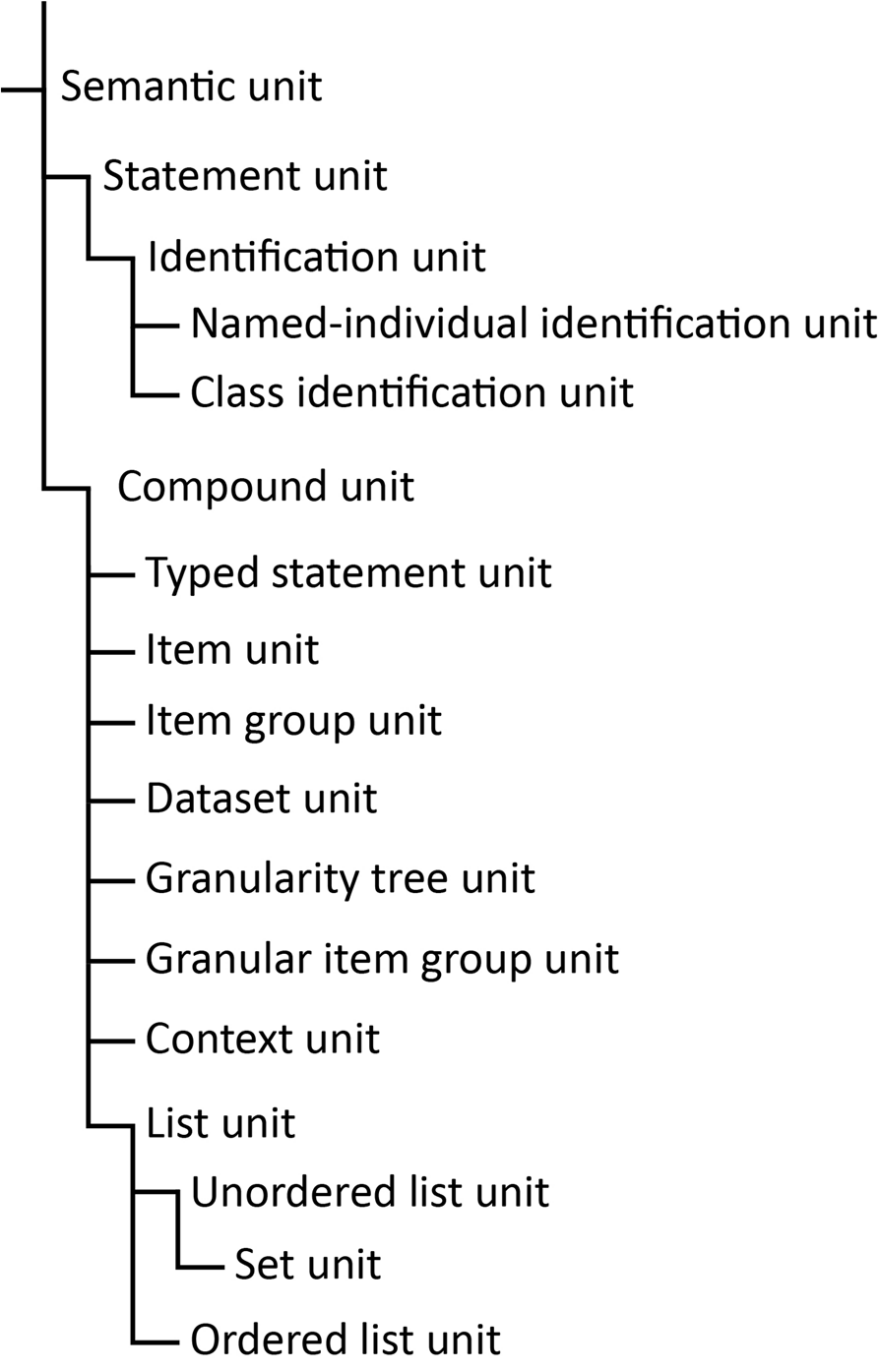
Classification of different categories of semantic units

Beyond statement units, which constitute smallest semantically meaningful statements (e.g., a weight measurement), collections of statement units can form compound units representing a coarser level of representational granularity. The classification of semantic units thus distinguishes two fundamental categories—**statement units** and **compound units**, each with its respective subcategories. For a detailed classification of semantic units, refer to Fig. 5.

**Fig. 6:**
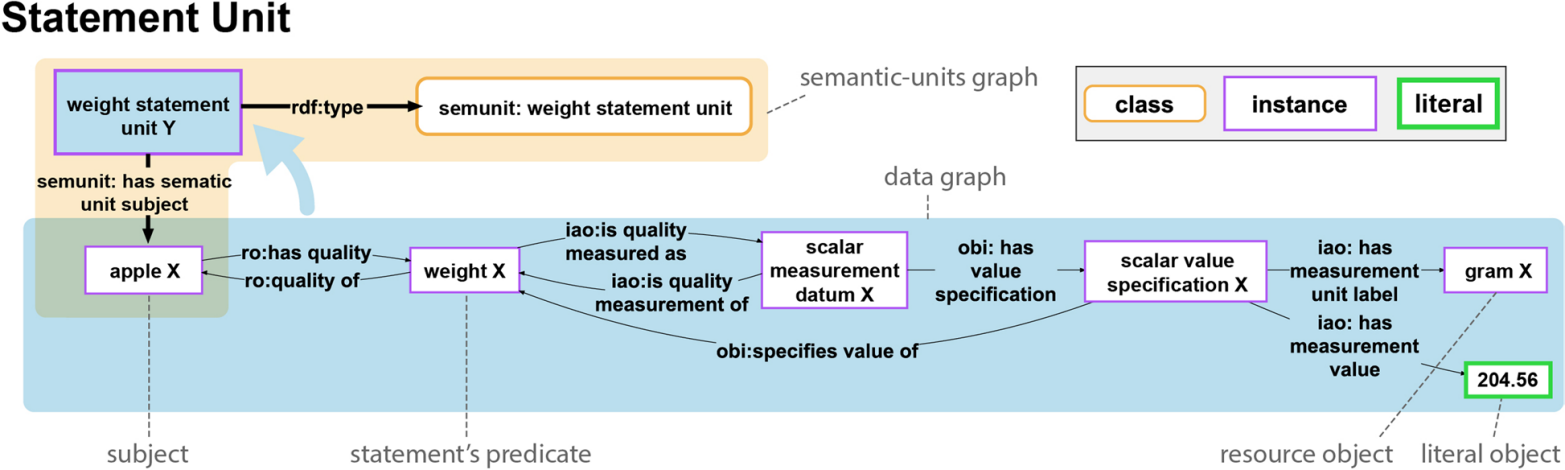
Example of a statement unit. The illustration displays a statement unit exemplifying a has-weight relation. The data graph, denoted within the blue box at the bottom, articulates the statement with ‘apple X’ as the subject and ‘gram X’ alongside the numerical value 204.56 as the objects. The peach-colored box encompasses the semantic-units graph, housing triples that encapsulate the semantic unit’s representation. It explicitly denotes the resource embodying the statement unit (bordered blue box), an instance of the *SEMUNIT:weight statement unit* class, with ‘apple X’ identified as the subject. Notably, the UPRI of *’weight statement unit’* is also the UPRI of the semantic unit’s data graph (the unbordered subgraph in the blue box)

The structuring of a knowledge graph into semantic units involves introducing an additional layer of triples to the existing graph. To distinguish these two layers, we label the pre-existing graph as the **data graph layer**, while the newly added triples constitute the **semantic-units graph layer**. For clarity across the graph, the resource representing a semantic unit, along with all triples featuring this resource in the *Subject* or *Object* position, is assigned to the semantic-units graph layer. Extending this distinction from the graph as a whole to individual semantic units, each semantic unit is associated with both a data graph and a semantic-units graph. The data graph of a particular semantic unit shares the same UPRI as its semantic unit resource. This alignment enables reference to the UPRI, concurrently denoting the semantic unit as a resource and its corresponding data graph. This interconnectedness empowers users to make statements about the content encapsulated within the semantic unit’s data graph, as shown in Fig. 6.

#### Statement unit: a proposition in the knowledge graph

A statement unit is characterized as the **fundamental unit of information** encapsulating the **smallest, independent proposition (i.e., statement) with semantic meaning for human comprehension** (see also [32]). For instance, the weight measurement statement for *apple X* illustrated in Fig. 6 represents a statement unit.

Structuring a knowledge graph into statement units results in a **partition of its graph**. Each triple within the data graph layer of the knowledge graph is associated with exactly one statement unit, and merging the subgraphs of all statement units results in the complete data graph of a knowledge graph. This partitioning only applies to the data graph layer.

We can understand each statement unit to specify a particular proposition by establishing a relationship between a resource serving as the subject and either a literal or another resource, denoted as the object of the predicate. Every statement unit encompasses a single subject and one or more objects.

To illustrate, a has-part statement unit features a subject and one object. Conversely, a weight measurement statement unit consists of a subject and two objects—the weight value and the weight unit (refer to Fig. 6). The resource signifying a statement unit in the graph establishes a connection with its subject through the property *SEMUNIT:hasSemanticUnitSubject*, which is documented in the semantic-units graph of the statement unit.

In scenarios where the proposition within the data graph is grounded in a **binary relation**—a divalent predicate like ‘*This right hand has as a part this right thumb*’—the associated statement unit typically comprises a single triple. This alignment arises from the nature of RDF, where *Predicates* of triples are inherently binary relations. In such cases, the RDF property concurrently embodies the statement’s verb or predicate. However, numerous propositions are grounded in **n-ary relations**, making a single triple insufficient for their representation. Examples encompass the weight measurement statement in Fig. 6 and statements like ‘*This right hand has part this right thumb on January 29*^*th*^* 2022*’, ‘*Anna gives Bob a book*’, and ‘*Carla travels by train from Paris to Berlin on the 29th of June 2022*’, each necessitating more than one triple. In these cases, the statement’s verb or predicate is often represented not by a property within a single triple but instead by an instance resource, as exemplified by ‘weight X’ (PATO:0000128) in Fig. 6. The composition of statement units, whether consisting of one or more triples, is contingent upon the relation of the underlying proposition, the n-aryness of its predicate, and the incorporation of optional objects. Types of statement units can be distinguished based on the n-ary verb or predicate that characterizes their underlying proposition. Notably, numerous object properties of the Basic Formal Ontology 2 denote ternary relations, particularly those entailing temporal dependencies [48]. For instance, ‘*b* located_in *c* at *t*’ mandates at least two triples for accurate representation in RDF.

The determination of which triples belong to a statement unit necessitates case-by-case specification by human domain experts. The statement unit patterns can then be specified using languages like LinkML [49, 50] or the Shapes Constraint Language SHACL [51]. These languages enable the definition of graph patterns to represent specific propositions, subsequently constituting a statement unit. Each statement unit instantiates a designated statement unit class, a classification defined by the specific verb or predicate characterizing the propositions modelled by its instances. We can distinguish different subcategories of statement units based on the underlying predicate, such as *has part*, *type*, *develops from*.

**Fig. 7:**
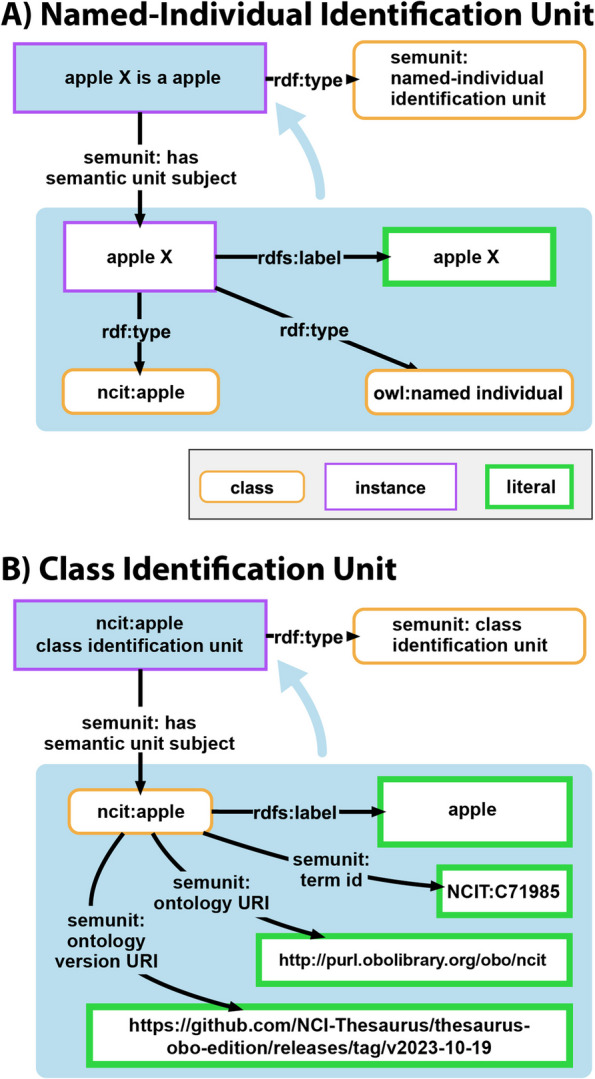
Examples for two different types of identification units. **A** **Named-individual identification unit**. The data graph within the unbordered box delineates the class-affiliation of the ‘apple X’ (NCIT:C71985) instance. The subject, ‘apple X’, is connected to its class through the property *type* (RDF:type), while its label ‘apple X’ is conveyed via the property  *label* (RDFS:label). The unbordered blue box designates the data graph associated with this named-individual identification unit. **B** **Class identification unit**. This data graph of this unit, represented by the unbordered blue box, captures the label and identifier of the class ‘apple’ (NCIT:C71985), the unit’s designated subject. Optionally, it includes the URI details of the ontology and the ontology version from which the class is derived. The bordered blue box designates the resource of this class identification unit

A distinctive category within the statement units, denoted as **identification units**, serves a specific purpose, providing details about a particular named individual or class resource. Two principal subtypes define this category. A **named individual identification unit** is a statement unit that serves to identify a resource to be a named individual, adding information such as the resource’s label, type, and its class membership (refer to Fig. 7A). A **class identification unit**^2^ is a statement unit that serves to identify a resource to be a class and provides details including its label, identifier, and optionally, the URIs of both the ontology and the specific version from which the class term has been imported (refer to Fig. 7B). Both types of identification units are important for providing human-readable displays of statement units, as they provide the labels for the resources used in them (see ‘typed statement unit’ and ‘dynamic label’ below).

#### Compound unit: a collection of propositions

Compound units are containers of collections of associated semantic units, each possessing semantic significance for a human reader.

**Fig. 8:**
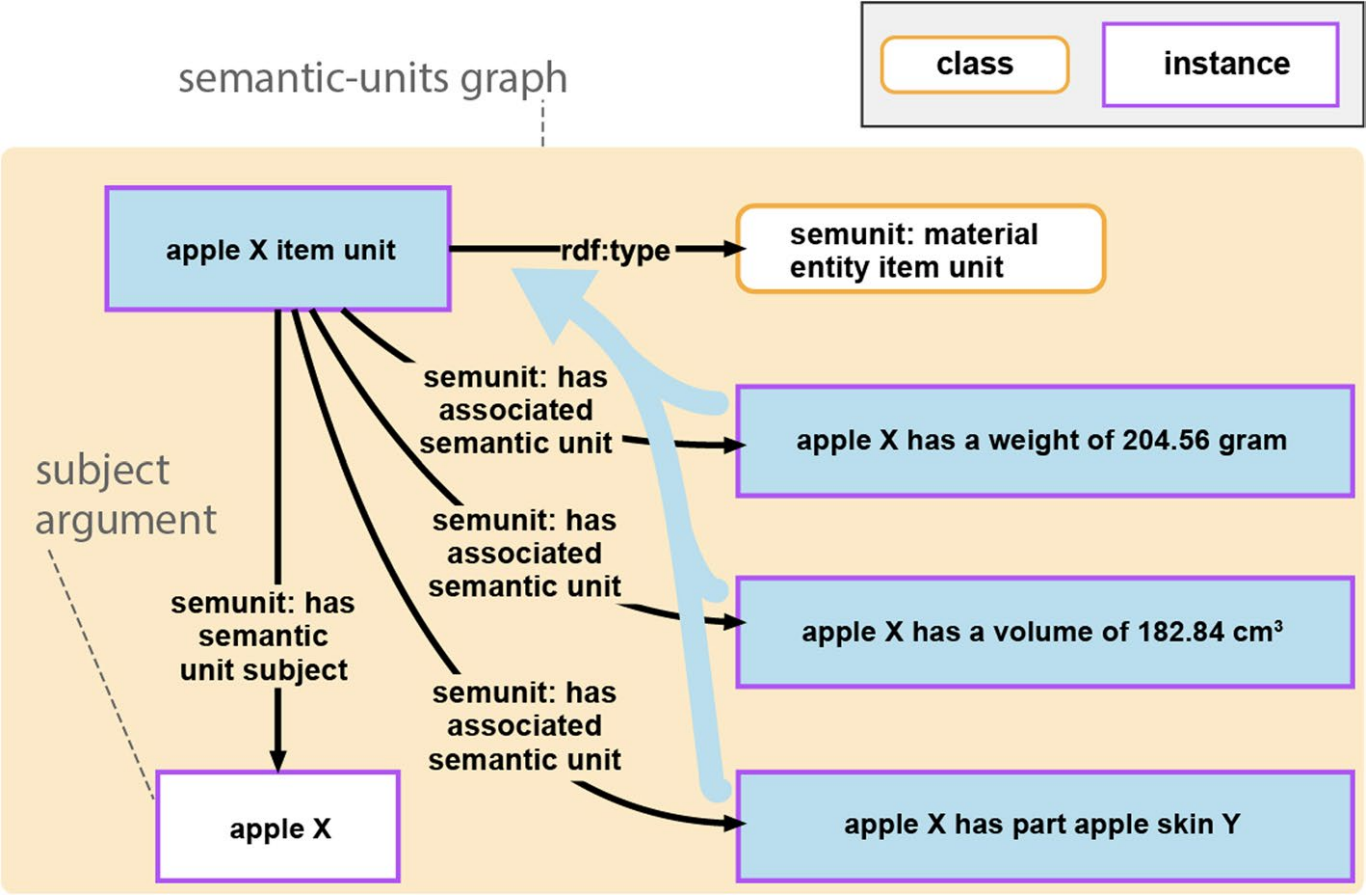
Example of a compound unit, denoted as *‘apple X item unit’*, that encompasses multiple statement units. Compound units, by virtue of merging the data graphs of their associated statement units, indirectly manifest a data graph (here, highlighted by the blue arrow). Notably, the compound unit possesses a semantic-units graph (depicted in the peach-colored box) delineating the associated semantic units

Each compound unit possesses a UPRI and instantiates a corresponding compound unit class. The connection between the resource representing the compound unit and those representing its associated semantic units is detailed through the property *SEMUNIT:hasAssociatedSemanticUnit* (see Fig. 8). The subsequent sections introduce distinct subcategories of compound units.

##### Typed statement unit

**Fig. 9:**
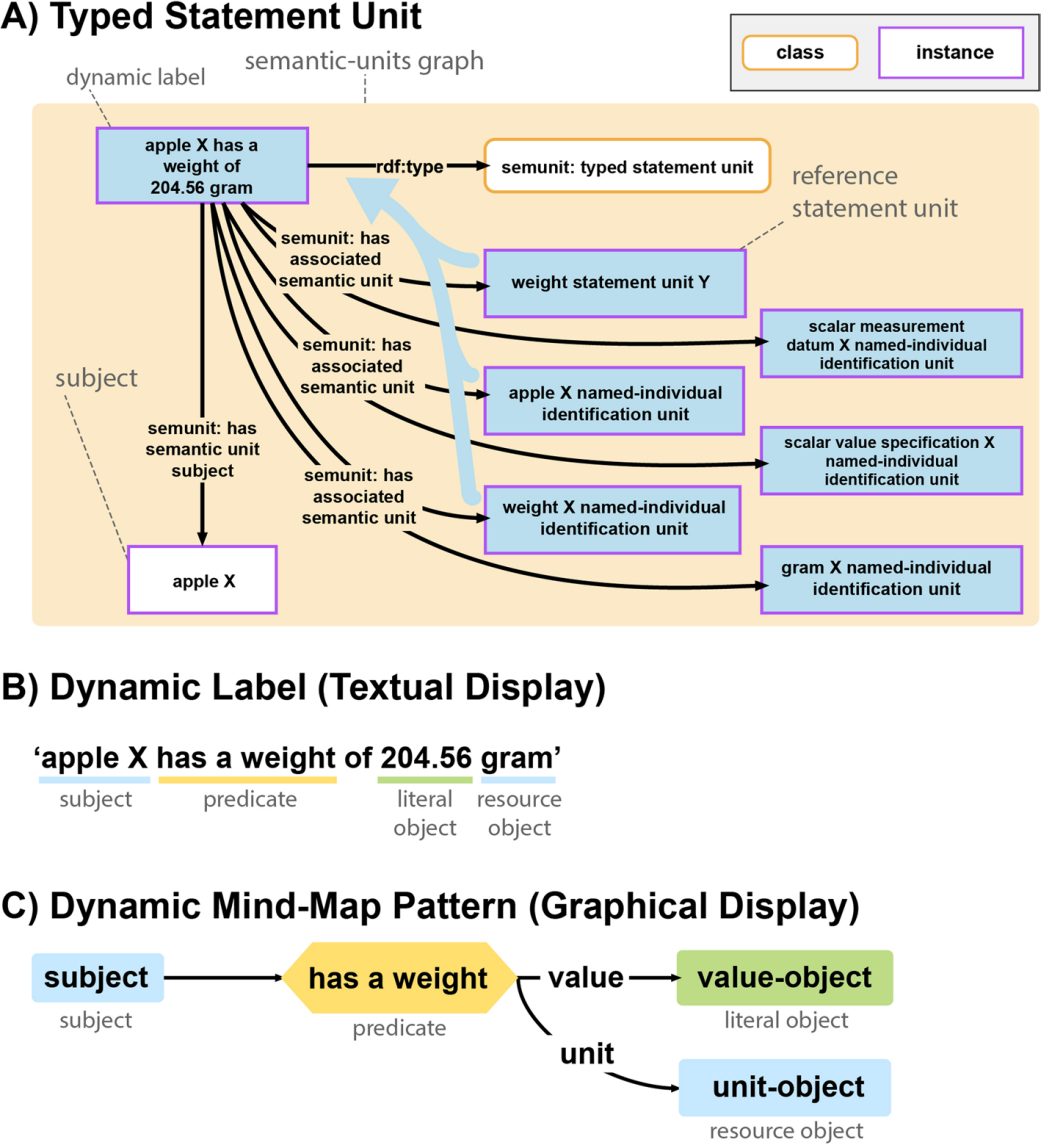
Typed statement unit with dynamic label and dynamic mind-map pattern. **A** **Typed statement unit** exemplified for a weight statement. This typed statement unit consolidates the data graphs of six statement units, including the *’weight statement unit’* from Figure 6, serving as the reference statement unit for this *‘typed statement unit’*, and five instances of *SEMUNIT:named-individual identification unit*. **B** **Dynamic label**: Illustrated is an example of the dynamic label associated with the reference statement unit class (*SEMUNIT:weight statement unit*). This dynamic label template is utilized for textual displays of information from the reference statement unit. **C** **Dynamic mind-map pattern**: Depicted is an example of the dynamic mind-map pattern associated with the reference statement unit class (*SEMUNIT:weight statement unit*). This pattern template is employed for graphical displays of information from the reference statement unit

A typed statement unit assigns a human-readable label to a statement unit. A typed statement unit is a compound unit comprising the following statement units (see Fig. 9A):

1. A statement unit that is not an instance of a named-individual or a class identification unit. It functions as the **reference statement unit** of the typed statement unit, and its subject is also the subject of the typed statement unit.
2. **Identification units** specifying the class affiliations of all the resources that are referenced in the data graph of the reference statement unit, together with their human-readable labels.

Each statement unit class has at least one display pattern associated with it. A display pattern acts as a template that takes as input the labels provided by the identification units associated with a typed statement unit and generates a **human-readable dynamic label** for the textual (see Fig. 9B) or a **dynamic mind-map pattern** for the graphical representation (see Fig. 9C) of the statement of its reference statement unit. Thus, a dynamic label and a dynamic mind-map pattern of a typed statement unit are derived from the corresponding templates provided by its reference statement unit, taking the human-readable labels provided by its identification units as input.

##### Item unit

An item unit encompasses all statement and typed statement units that share a common subject, i.e., they form a group of statements relating to the same entity. The subject resource becomes the subject of the item unit, and the resource representing an item unit in the semantic-units graph relates to its subject through the property *SEMUNIT:hasSemanticUnitSubject*. Conceptually, item units align with the *graph-per-resource* data management pattern [52] or the previously mentioned *characteristic set* or *RDF molecule*, and they are akin to the *Item* [https://www.mediawiki.org/wiki/Wikibase/DataModel#Item] concept in the Wikibase data model, but adapt the concept to statement units rather than triples.

##### Item group unit

An item group unit is composed of a minimum of two item units. The subgraphs of the item units belonging to the same item group unit are connected through statement units that share their subject with the subject of one item unit and one of their objects with the subject of another item unit. As a result, merging the subgraphs of all the item units of an item group unit forms a connected graph.

##### Granularity tree unit

We can further identify types of statement units that depend on **partial order relations** (i.e., relations that are transitive, reflexive, and asymmetric), forming partial orders. Examples include class-subclass relations in ontologies, parthood relations in descriptive statements, and sequential relations like *before* (RO:0002083) in process specifications. Partial order relations give rise to granular partitions that form **granularity trees** [53–55] and contribute to defining **granularity perspectives** [56–58].

Granularity perspectives identify specific types of semantically meaningful tree-like subgraphs within a knowledge graph, supporting graph exploration by modularization in addition to statement, item, and item group units.

Due to the nested structure of a granularity tree and its inherent directionality from root to leaves, the subject of a granularity tree unit can be specified as the subject of statement units sharing objects with the subjects but not their subject with the objects of other statement units within the same granularity tree unit.

##### Granular item group unit

A granular item group unit encompasses all statement units and item units whose subjects belong to the same granularity tree unit. The item units belonging to a granular item group unit can be systematically arranged within a nested hierarchy dictated by the underlying granularity tree. This additional organization offers improved **explorability** for users of a knowledge graph application.

##### Context unit

The *isAbout* property (IAO:0000136) connects an information artifact to an entity about which the artifact provides information. Using this property in a knowledge graph changes the frame of reference from the discursive layer to the ontological layer. An is-about statement thus divides a knowledge graph into two subgraphs, each forming a context unit that belongs to one of these two layers. Is-about statement units relate resources from the semantic-units graph with resources from the data graph of a knowledge graph. For example, in documenting a research activity that results in the creation of a dataset describing the anatomy of a multicellular organism, the statement *‘description item unit’* *isAbout* ‘multicellular organism’ (UBERON:0000468) marks a transition in the frame of reference from the research activity’s outcome to the multicellular organism being described (see also Fig. 12 further below).

##### Dataset unit

A dataset unit is an ordered set of semantic units. They can be employed to aggregate all data contributed by a specific institution in a collaborative project, document the state of a particular object at a given time, or store and make accessible the results of a specific search query. Knowledge graph users have the flexibility to specify dataset units for their individual needs, utilizing the unit’s UPRI as reference identifier.

##### List unit

In certain instances, it becomes necessary to articulate statements about a specific collection of particular resources. To achieve this, such a collection can be modelled as a list unit. We distinguish **unordered list units** from **ordered list units**, with the latter organizing resources in a specific sequence, such as the authors of a scholarly publication. Conversely, a **set unit** is an unordered list unit where each resource is listed only once, adhering to a uniqueness restriction.

From a technical standpoint, a list unit contains membership statement units, each delineating a resource belonging to the list by linking the UPRI of the list unit through a *SEMUNIT:child* relation to the respective resource. In the case of an ordered list unit, each membership statement unit must be indexed through a data property *index* (RDF:index).

List units can be employed as arrays and may incorporate cardinality restrictions, thereby characterizing a closed collection of entities and enabling a localized closed-world assumption.

## Discussion

### Benefits of organizing a knowledge graph into semantic units

#### Enhancing data management flexibility through modularity

The organization of a knowledge graph into distinct subgraphs, each associated with a particular semantic unit, introduces modularity in a graph. Each semantic unit, represented in the graph by a dedicated resource classified as an instance of a specific semantic unit class, serves as a **structured module that encapsulates complexity**. This modular approach allows for the **e**ncapsulation of subgraphs, and may add flexibility in data management as larger parts of a graph can be manipulated jointly.

**Semantic units operate at a higher level of abstraction than individual triples**. Semantically, they encapsulate the contents of their data graphs, representing statements or sets of semantically and ontologically related statements.

The specification of relations between semantic units further extends the flexibility of data management. A given semantic unit from a finer level of representational granularity can be associated with multiple units from a coarser level. Consequently, a statement unit may be linked to more than one compound unit, all while maintaining the centrality of the statement unit itself and its triples in a single location within the graph.

The modular nature introduced by semantic units may streamline partitioned-based querying of knowledge graphs. While other approaches for graph partitioning have shown success [59], employing semantic units for partitioning and establishing modularity in the graph is an avenue for future research exploration.

##### Semantic units as a framework for knowledge graph alignment

The instantiation of semantic units belonging to the same class inherently implies a semantic similarity across instances. This characteristic lays the groundwork for a systematic approach to aligning and comparing knowledge graphs that share a common set of semantic unit classes. The alignment process could operate in a stepwise manner across various levels of representational granularity. In the initial step, alignment focuses on item group units, leveraging their types of associated item units and their alignment for comparison. The latter alignment hinges on the types of subjects and the types of associated statement units, allowing for further alignment based on class. Ultimately, individual triples within the aligned statement units undergo comparison, marking a comprehensive strategy to enhance existing methods for knowledge graph alignment, subgraph-matching, graph comparison, and graph similarity measures.

##### Managing restricted access to sensitive data

The classification of statement units into corresponding ontology classes may serve as a framework for identifying subgraphs within a knowledge graph housing sensitive data that warrants restricted access. By identifying statement units containing sensitive information by class, access restrictions can be dynamically enforced based on specific criteria.

### Semantic units: A framework for nested and overlapping knowledge graph modules

#### Semantic units identify five levels of representational granularity

**Fig. 10:**
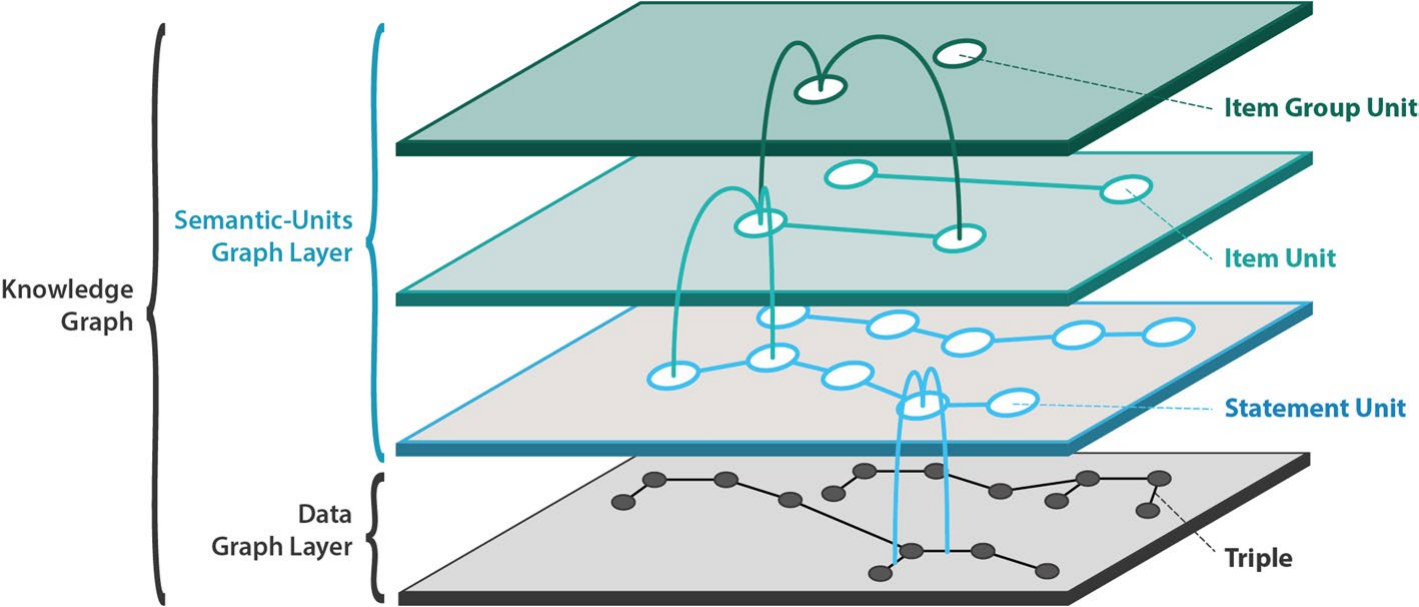
Five levels of representational granularity**.** The integration of semantic units into a knowledge graph introduces a semantic-units graph layer, enriching the existing data graph layer. This augmentation includes distinct levels, namely triples, statement units, item units, and item group units, providing a nuanced hierarchy of representational granularity within a knowledge graph

Semantic units introduce a structured framework encompassing **five levels of representational granularity** within a knowledge graph: triples, statement units, item units, item group units, and the knowledge graph as a whole (refer to Fig. 10). While triples represent the lowest level of abstraction, semantic units provide coarser levels, organizing the semantic-units graph layer (i.e., the **discursive layer of a knowledge graph**) and, indirectly, the knowledge graph’s data graph layer.

The hierarchical organization of triples into statement units (→ smallest units of propositions that are semantically meaningful for a human reader), further into item units (→ comprising all the information from the knowledge graph about a particular entity), and eventually into item group units (→ collections of semantically interrelated entities) could enhance human readability and usability. This structural hierarchy supports users in seamlessly navigating across the graph, zooming in and out of different levels of representational granularity.

#### Semantic units identify granularity trees

Granularity trees offer a perspective that is orthogonal to representational granularity, structuring the data graph layer and thus the **ontological layer of a knowledge graph** into **distinct granularity perspectives**. Consider the example of a multicellular organism’s description including a has-part statement unit stating that the organism has a head as its part. This unit is associated with the item unit of the organism itself, which is linked to additional item units about the organism’s other parts, constituting an item group unit. Moreover, since has-part is a partial order relation [55], the has-part statement unit is associated with a parthood granularity tree unit and its corresponding granular item group unit. Consequently, the statement unit is associated with at least four different compound units that can be communicated to the user alongside the statement itself, showcasing the versatility enabled by semantic units in exploring contextualized subgraphs [54].

### Semantic units identify context-dependent subgraphs

Semantic units empower the organization of item group units into context units, each defining a specific frame of reference. Intersections between context units are discerned through is-about statements (see also Fig. 12), facilitating traversal across diverse frames of reference. Context units contribute to structuring the data graph layer and thus the ontological layer of a knowledge graph into different **frames of reference**.

#### Statements about statements and documenting ontological and discursive information in knowledge graphs using semantic units

The introduction of semantic units provides a framework for making **statements about statements** in a knowledge graph. Each semantic unit, equipped with its unique UPRI and represented in the semantic-units graph layer, facilitates **assertions about statement units**. This structured approach offers the potential for cross-database and cross-knowledge-graph statements when semantic units are implemented as nanopublications or FAIR Digital Objects, addressing the challenge of making statements about statements in knowledge graphs.

**Fig. 11:**
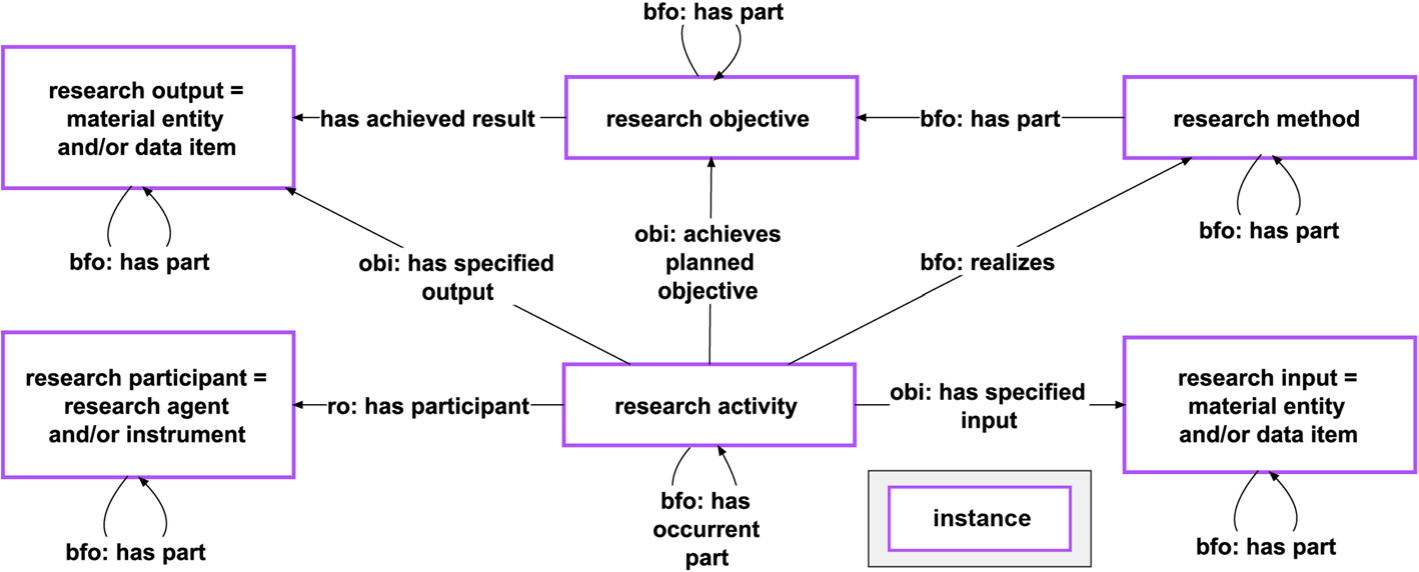
A semantic schema for modelling the contents of scholarly publications. The depicted semantic schema outlines the modelling structure for encapsulating the components of scholarly publications. It delineates the relationship between a research activity, its associated input and output, and the underlying specification of its process plan, manifested in the form of a research method and research objective. The model draws inspiration from [61]

Moreover, if a knowledge graph should cover contextual assertions such as “*Author A asserts that the melting point of lead is at 327.5 °C*” or “*The assertion about the melting point of lead being at 327.5 °C is a result of experiment X”*, it becomes challenging to model this without having a formalism for representing such **discursive contextual information** and its relationship to empirical data (see also Ingvar Johannson’s distinction between *use* and *mention* of linguistic entities [60]). Statement units with their data graphs contribute **ontological information**, nested within compound units of coarser representational granularity. In the semantic-units graph, propositions are represented as nodes, forming a significant portion of the **discursive layer**. Additionally, context units allow the explicit documentation of **different frames of reference** within both the ontological and discursive layers. The ability of statement units to establish relations between resources or even between other statement units (e.g., ‘*author_A -asserts-> statement_unit_Y*’; ‘*statement_unit_X -hasMetadata-> statement_unit_Z*’) facilitates the documentation of connections between the empirical and discursive layers. For instance, an item group unit focusing on the contents of a scholarly publication, can encapsulate information about the associated research activity, its inputs, outputs, research methods, and objectives (see Fig. 11).

**Fig. 12:**
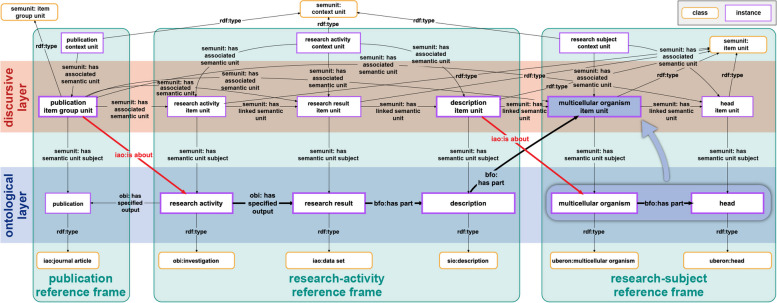
Detail from the RDF graph illustrating the contents of a scholarly publication. The data schema employed aligns with the schema shown in Figure 11, tailored to accommodate semantic units. The publication’s content is encapsulated within a dedicated publication item group unit instance through various interconnected semantic units. The publication itself is denoted as an instance of journal article (IAO:0000013). The publication item group unit encompasses multiple item units related to the research activity, interconnected through the *SEMUNIT:hasLinkedSemanticUnit* property. The interconnected hierarchy extends to an investigation (OBI:0000066) instance, resulting in a data set (IAO:0000100) instance with a description (SIO:000136) instance as its part. This description, in turn, has the multicellular organism item unit describing the organism as its part, which has an instance of multicellular organism (UBERON:0000468) as its subject. The blue arrow signifies the representation of the data graph (dark blue box with shadow) by this specific item unit (bordered box in the same color). The ontological layer is constituted by the data graphs of the semantic units, while their semantic-units graphs collectively form the discursive layer. Distinct context units demarcate the reference frames of the publication, research-activity, and research-subject, delineated by is-about statements. *For reasons of clarity of presentation, the associated statement units are not shown in the discursive layer*

The proposed model may find application within a knowledge graph centered around scholarly publications. For example, the representation in Fig. 12 combines the discursive and the ontological layers and represents the connections between different frames of reference.

### Implementation

#### Implementing semantic units in RDF/OWL-based knowledge graphs using Nanopublications

To initiate the structuring of a knowledge graph into semantic units, first, a layer of abstraction beyond the triple level must be created. This is accomplished by partitioning the knowledge graph into a set of statement units, where each triple belongs exclusively to one data graph of a statement unit. In RDF/OWL, statement units can be conceptualized like nanopublications.

**Nanopublications** are RDF graphs that serve as the smallest published information units extracted from literature and enriched with provenance and attribution information [62–65]. Leveraging Named Graphs and Semantic Web technologies, each nanopublication models a particular assertion, such as a scientific claim, in a machine-readable format and semantics and is accessible and citable through a unique identifier. Each nanopublication is organized into four Named Graphs:

1. the *head* Named Graph, connecting the other three Named Graphs to the nanopublication’s unique identifier;
2. the *assertion* Named Graph, containing the assertion modelled as a graph;
3. the *provenance* Named Graph, containing metadata about the assertion; and
4. the *publicationInfo* Named Graph, containing metadata about the nanopublication itself.

The *assertion* Named Graph would contain the data graph of a statement unit, whereas the *head* Named Graph its semantic-units graph. Triples in the *provenance* Named Graph can potentially link to other semantic units and thus other nanopublications that contain detailed metadata descriptions (e.g., a metadata graph as shown in Fig. 4).

A compound unit, being a collection of two or more semantic units, can be organized in an RDF/OWL-based knowledge graph by linking the compound unit’s UPRI to the UPRIs of its associated semantic units. Following the nanopublication schema, this can be implemented by employing the compound unit’s semantic-units graph as the *head* Named Graph of a corresponding nanopublication, leaving the nanopublication’s *assertion* Named Graph empty. The *head* Named Graph thus specifies all statement and compound units associated with this compound unit.

#### Implementing semantic units in Neo4j-based knowledge graphs using UPRIs and corresponding property-value pairs

In Neo4j, a labeled property graph, the assignment of UPRIs to all nodes and relations through a ‘*UPRI:upri*’ property-value pair is an essential prerequisite for implementing semantic units. To identify all triples affiliated with the same statement unit, a ‘*statement_unit_UPRI:upri*’ property-value pair must be added to each node and relation belonging to the statement unit, with the statement unit’s UPRI serving as the value. Building on this primary abstraction layer of statement units, a secondary abstraction layer of compound units can be organized. The nodes and relations associated with all triples within a compound unit are endowed with a ‘*compound_unit_UPRI:upri*’ property-value pair, having the compound unit’s UPRI as their value. Since a particular statement unit may be associated with multiple compound units, its ‘*compound_unit_URI*’ property can incorporate an array of UPRIs representing different semantic units.

**Fig. 13:**
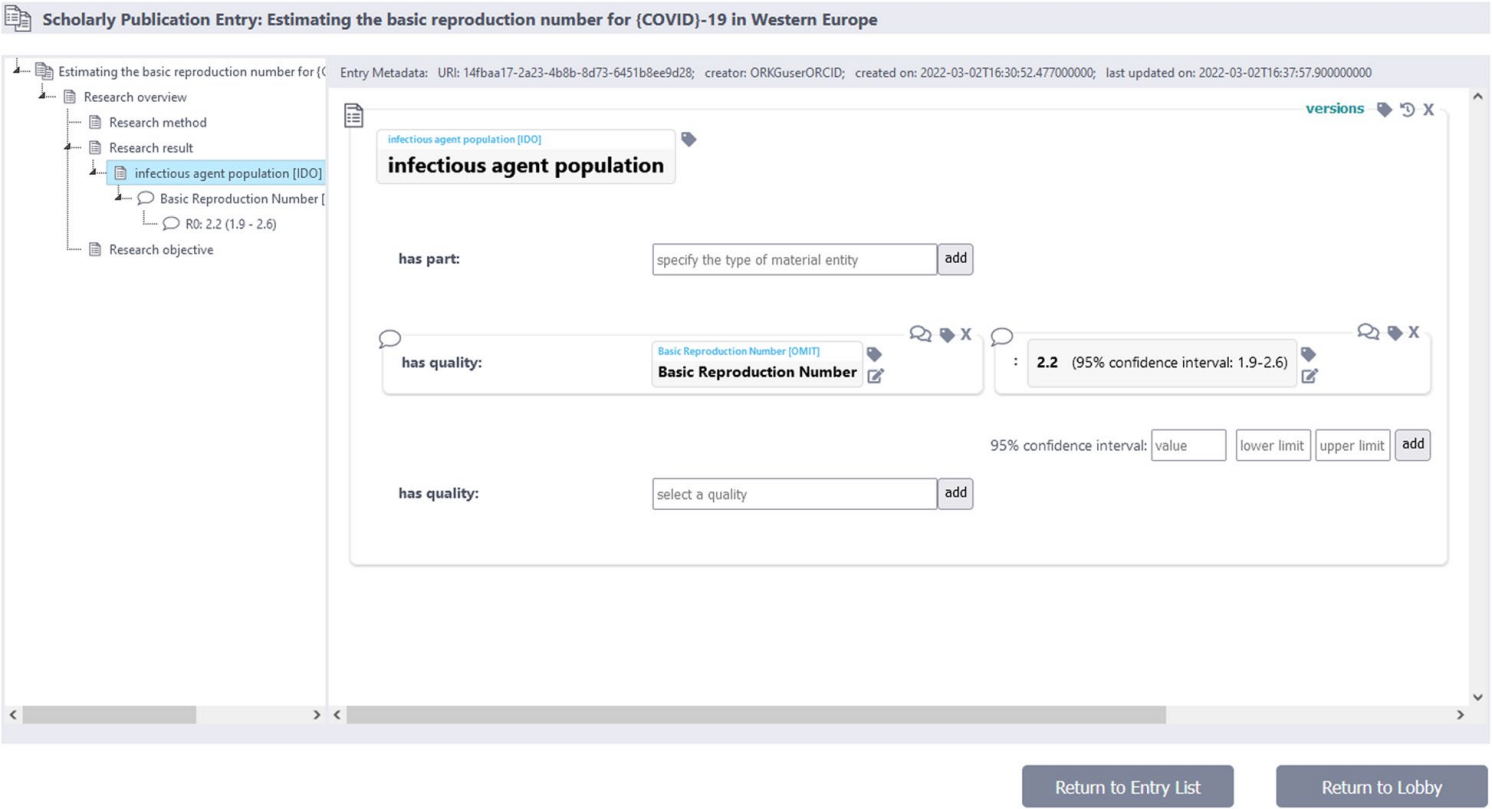
User interface of a prototype web application that implements semantic units. On the left is a navigation tree that leverages the organization of the underlying Neo4j knowledge graph into different item group, item, and statement units. Currently selected is the infectious agent population item group. On the right, all statements belonging to the selected item group are displayed

An initial software for demonstration purposes has been developed by one of the authors, illustrating how semantic units can manage a knowledge graph [66]. Built upon Neo4j as the persistence-layer technology, the application sources its content via a web interface and user input. This small-scale knowledge graph application is designed for documenting assertions from scholarly publications, offering users an exemplary platform to describe some of the contents (and not merely bibliographic metadata) found in a scholarly publication. Each described paper stands as its own item group unit, featuring assertions covered by statement units linked to item units and granularity tree units. The prototype encompasses versioning of semantic units and automatic tracking of their editing histories and provenance. The application employs the organization of the graph into semantic units within a navigation tree, facilitating exploration of a given item group unit through its associated item units (see Fig. 13). The showcase is built using Python and flask/Jinja2 and is openly available at https://github.com/LarsVogt/Knowledge-Graph-Building-Blocks.

#### Strategies for implementation

Given that only statement units store information, while compound units act as their containers, the first step of implementing semantic units should focus on identifying the statement unit classes required for representing the types of statements integral to the knowledge graph’s coverage. Each statement unit class requires an assigned graph schema, preferably articulated using a shapes constraint language like [51] SHACL. In this initial step, statement types that are grounded in partial order relations must be identified as well (required for identifying granularity tree units). From here, three distinct implementation strategies are available:

1. **Develop from scratch**: In cases where no knowledge graph exists yet, the focus should be on developing a knowledge graph application that organizes incoming information into statement units in accordance with their assigned graph schemata. Rules for organizing statement units into compound units, contingent on the compound unit type, must be established. For example, statement units sharing the same subject resource form a corresponding item unit.
2. **Transfer an existing knowledge graph**: If there is an existing knowledge graph that needs restructuring into semantic units, crafting queries to transfer all triples into corresponding statement units, based on the graph schemata identified in the first step, is the next step. The main challenge is maintaining disjointedness of triples between statement units.
3. **A hybrid approach**: For scenarios where restructuring an entire knowledge graph seems impractical or undesirable, but there is a desire to organize newly added information into semantic units, a hybrid approach is possible. This involves developing input workflows to ensure that all incoming data conforms to the semantic units structure.

#### Semantic Units as FAIR Digital Objects

The concept of **FAIR Digital Objects**, as proposed by the European Commission Expert Group on FAIR Data, stands at the core of achieving the FAIR Principles [67], emphasizing persistent identifiers, comprehensive metadata, and contextual documentation for reliable discovery, citation, and reuse. The concept of semantic units aligns with that of FAIR Digital Objects. Each semantic unit inherently possesses a UPRI, serving as a ready-made persistent identifier. Accessibility and searchability are ensured through established protocols like SPARQL and CYPHER, with RDF, JSON, and other formats supporting data export. When knowledge graphs adhere to controlled vocabularies and ontologies, and when they employ standard graph-patterns using tools like SHACL [51], ShEx [68, 69], or OTTR [70, 71], the data within the data graphs of semantic units may more easily achieve semantic interoperability.

Moreover, semantic units can provide provenance—crucial for tracking a semantic unit’s history—through utilizing property-value pairs for labeled property knowledge graphs or a designated provenance Named Graph for RDF/OWL knowledge graphs. The **provenance metadata** of a semantic unit encompasses details like the creator, creation date, application used, title, contributing users, and last-update—focusing solely on the semantic unit itself, not the original data production process.

**Access control metadata** can specify any licenses as well as access control restrictions.

## Conclusion and future work

In conclusion, the adoption of semantic units in structuring knowledge graphs may be useful to address the challenges faced in knowledge representation mentioned in the introduction. By encapsulating each statement within its dedicated statement unit, accompanied by a corresponding statement unit class and **data schema** (e.g., as a SHACL shape), a robust foundation for FAIR data and metadata is established supporting **schematic interoperability**. Because statement units partition the knowledge graph so that every triple belongs to exactly one statement unit and every statement unit’s subgraph is identifiable and referenceable through its UPRI, data in a knowledge graph is linked to graph patterns which are identifiable as a whole. By providing each schema its own UPRI, each semantic unit can specify its underlying schema in its metadata. Identifying semantically interoperable semantic units is then straightforward, and schema crosswalks between different schemata can increase schematic interoperability [72] (*Challenge 1*).

Graph query languages can use the graph patterns (semantic units), and therefore allow access to knowledge graph content through higher levels of abstractions than basic triples (*Challenge 2*).

Further, we have shown how semantic units can organize knowledge graphs in different layers and make **statements about statements** (*Challenge 3*).

Future research involves extending the semantic units approach to incorporate question units and a nuanced categorization of assertional, contingent, prototypical, and universal statement units. This extension will encompass formal semantics for the latter, including provisions for negations and cardinality restrictions. Additionally, we are exploring novel approaches to knowledge graph exploration based on semantic units.

## Data Availability

Not applicable.

## Abbreviations

BFO: Basic Formal Ontology
CRUD: Create, Read, Update, Delete
FAIR: Findable, Accessible, Interoperable, and Reusable
HTTP: Hypertext Transfer Protocol
HTTPS: Hypertext Transfer Protocol Secure
IAO: Information Artifact Ontology
ID: Identifier
JSON: JavaScript Object Notation
LinkML: Linked Data Modeling Language
NCIT: National Cancer Institute
NoSQL: Not only Structured Query Language
OBI: Ontology for Biomedical Investigations
OBOE: Extensible Observation Ontology
OBO Foundry: Open Biological and Biomedical Ontology Foundry
OTTR: Reasonable Ontology Templates
OWL: Web Ontology Language
PATO: Phenotype and Trait Ontology
RDF: Resource Description Framework
RDFS: RDF-Schema
RO: OBO Relations Ontology
SHACL: Shape Constraint Language
ShEx: Shape Expression
SIO: Semanticscience Integrated Ontology
SPARQL: SPARQL Protocol and RDF Query Language
TI: Time Ontology in OWL
TRUST: Transparency, Responsibility, User Focus, Sustainability, and Technology
UBERON: Uber-anatomy ontology
UO: Units of Measurement Ontology
UPRI: Unique Persistent and Resolvable Identifier
XSD: Extensible Markup Language Schema Definition

## Footnotes

1 Machine-actionable are data and metadata that are machine-interpretable and that belong to a type for which operations have been specified in symbolic grammar, such as logical reasoning based on description logics for statements formalized in the Web Ontology Language (OWL) or rule-based data transformations such as unit conversion for defined types of elements [73].

2 Analog to class identification units, one could specify property identification units that have property resources as their subject.
