# Correction to: Semantic units: organizing knowledge graphs into semantically meaningful units of representation

**DOI:** 10.1186/s13326-024-00313-2

**Published:** 2024-06-06

**Authors:** Lars Vogt, Tobias Kuhn, Robert Hoehndorf

**Affiliations:** 1 TIB Leibniz Information Centre for Science and Technology, Welfengarten 1B, 30167 Hanover, Germany; 2 Department of Computer Science, Vrije Universiteit, Amsterdam, Netherlands; 3 Computational Bioscience Research Center, Computer, Electrical and Mathematical Sciences & Engineering Division, King Abdullah University of Science and Technology, 4700 KAUST, Thuwal, 23955 Saudi Arabia

**Correction to: Journal of Biomedical Semantics (2024) 15:7**

10.1186/s13326-024-00310-5

Following publication of the original article [1], we have been notified that the authors’ first and last names were switched and published incorrectly.

It is now:

Vogt Lars1*, Kuhn Tobias2 and Hoehndorf Robert3

It should be:

Lars Vogt1*, Tobias Kuhn2 and Robert Hoehndorf3
